# Supplementary figures and images for: Global organization of neuronal activity only requires unstructured local connectivity
Source: eLife. 2022 Jan 20;11:e68422. doi: 10.7554/eLife.68422 (PMC8776256; doi:10.7554/eLife.68422)

**A**

Utah array  
(motor cortex)

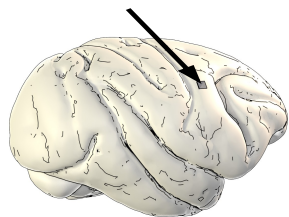**C**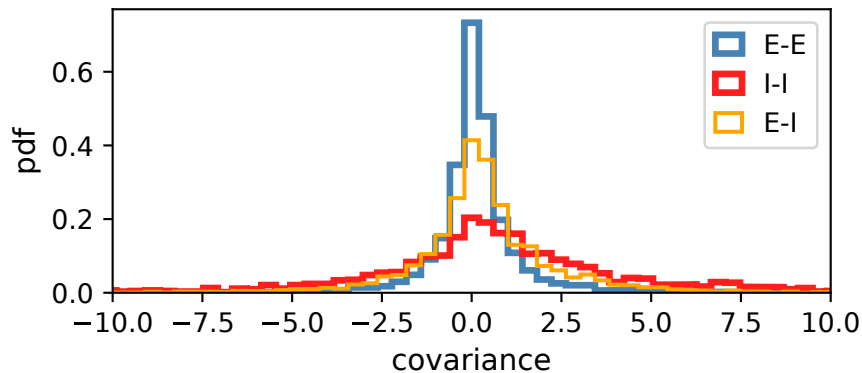**B**

Spike trains

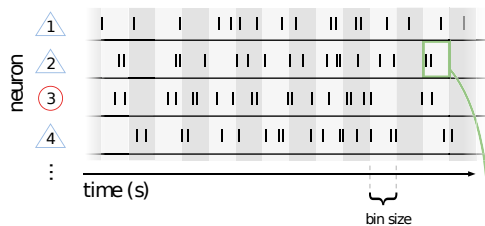

Binning

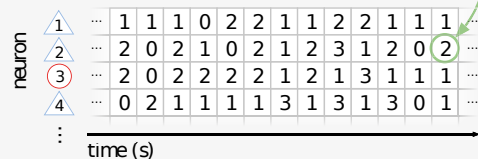**D**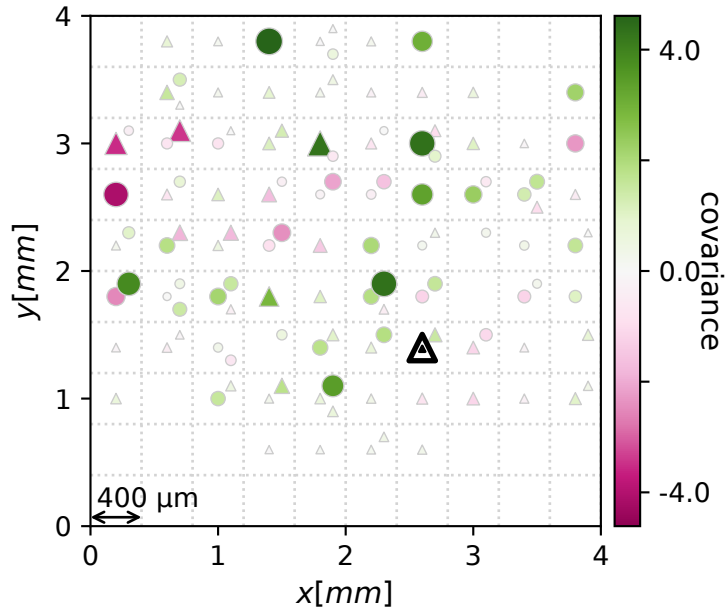

Supplement: Figure 1—source data 1. [file elife-68422-fig1-data1.zip › fig1/plots/figure1_E2.pdf]

**A**

Utah array  
(motor cortex)

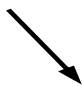**C**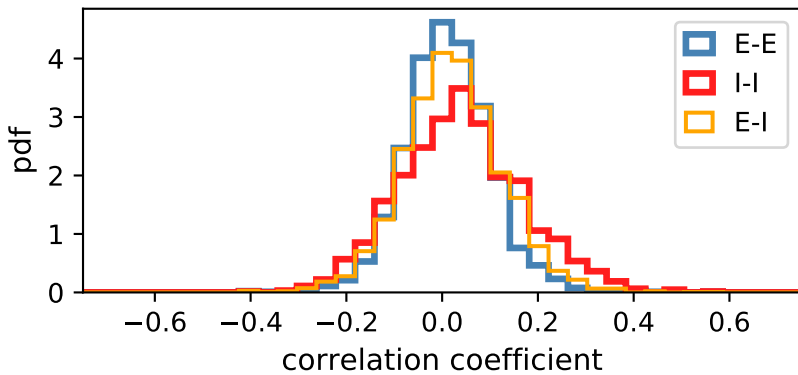**B****D**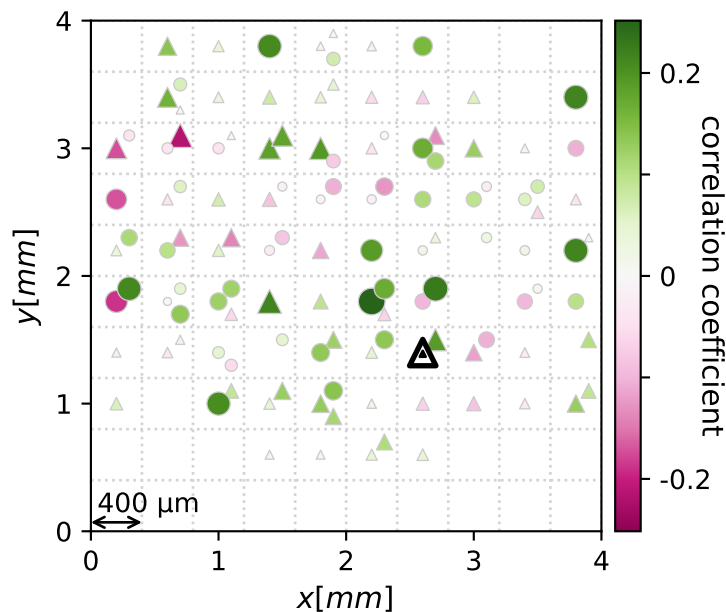

Supplement: Figure 1—source data 1. [file elife-68422-fig1-data1.zip › fig1/plots/fig1_E2.pdf]

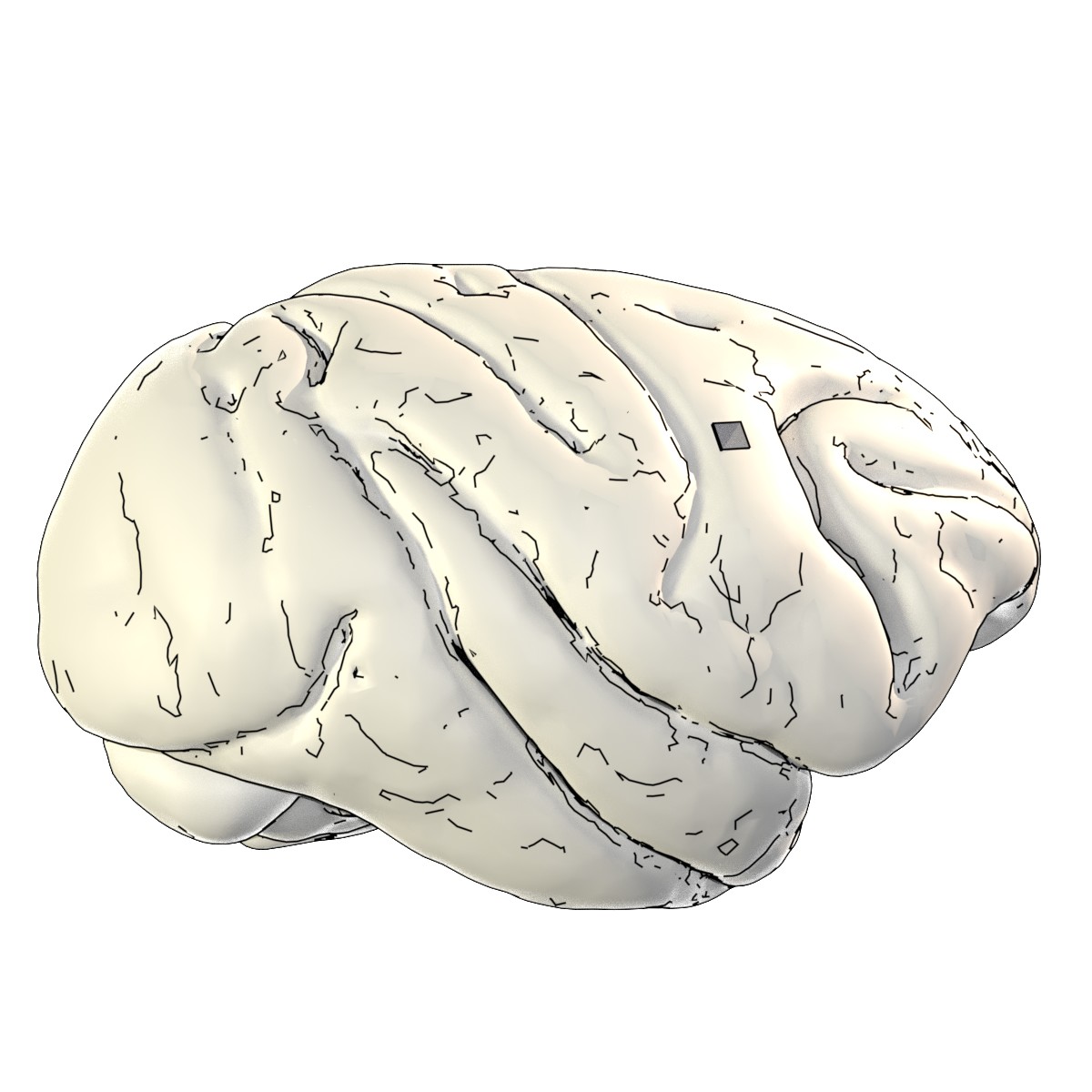

Supplement: Figure 1—source data 1. [file elife-68422-fig1-data1.zip › fig1/plots/Macaque-Brain-with-Utah-Array-countour-1200px.jpg]

**A**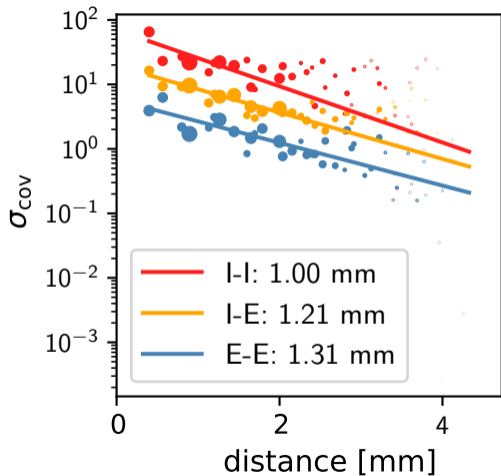**B**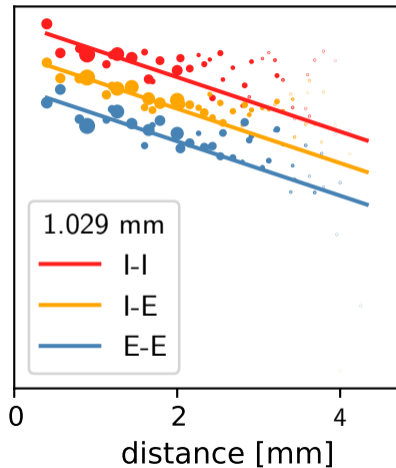**C**

| recording session | fitted decay constant [mm] | error <sub>b</sub> /error <sub>a</sub> |
|-------------------|----------------------------|----------------------------------------|
| E1                | 1.674                      | 1.1157                                 |
| E2                | 1.029                      | 1.0055                                 |
| N1                | 1.676                      | 1.0097                                 |
| N2                | 4.273                      | 1.0049                                 |

Supplement: Figure 4—source data 1. [file elife-68422-fig4-data1.zip › fig4/plots/figure4_E2.pdf]

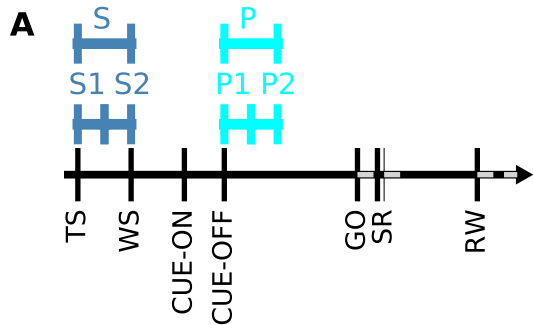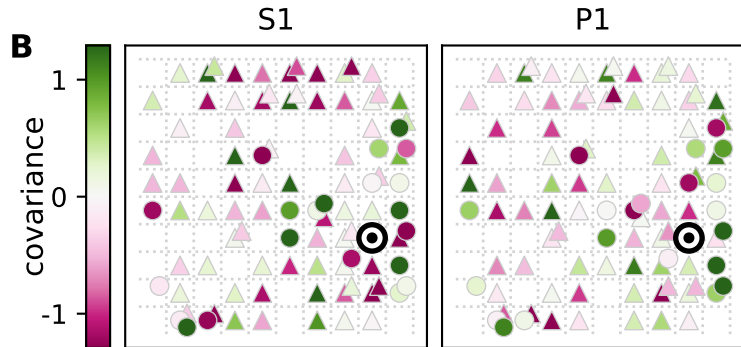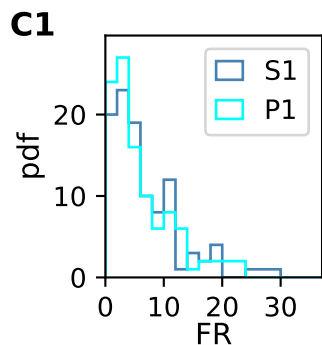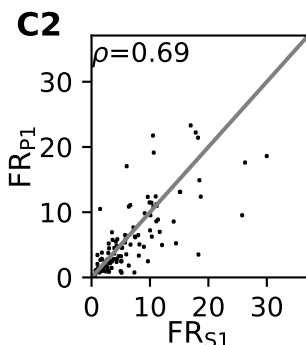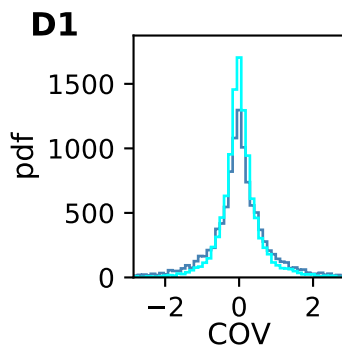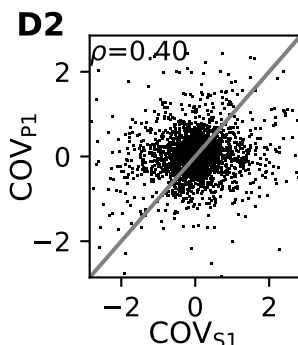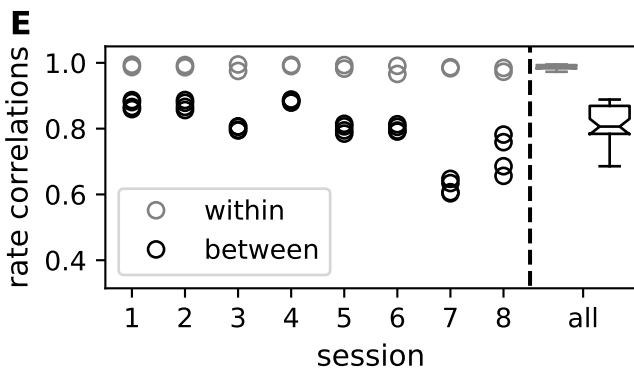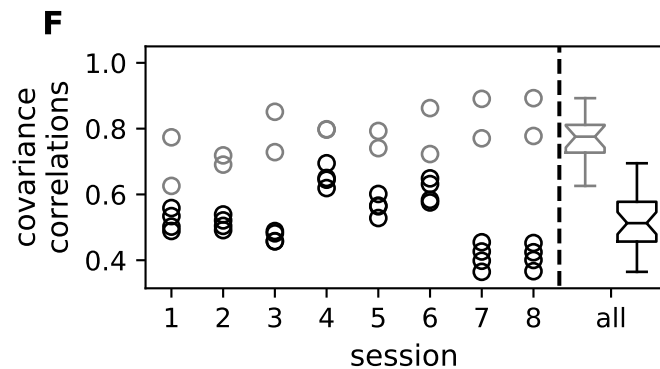

Supplement: Figure 6—source data 1. [file elife-68422-fig6-data1.zip › fig6/plots/fig6_i140725-002.pdf]
